# Assessing agricultural gene editing regulation in Latin America: an analysis of how policy windows and policy entrepreneurs shape agricultural gene editing regulatory regimes

**DOI:** 10.3389/fbioe.2023.1209308

**Published:** 2023-06-09

**Authors:** Sebastian Zarate, Ilaria Cimadori, Michael S. Jones, Maria Mercedes Roca, S. Kathleen Barnhill-Dilling

**Affiliations:** ^1^ Genetic Engineering and Society Center, North Carolina State University, Raleigh, NC, United States; ^2^ Forestry and Environmental Resources, North Carolina State University, Raleigh, NC, United States; ^3^ Yale School of the Environment, Yale University, New Haven, CT, United States; ^4^ Institute of Social and Economic Research, University of Alaska Anchorage, Anchorage, AK, United States; ^5^ BioScience Thinktank, Guadalajara, México

**Keywords:** gene editing, Latin America, policy, regulation, agricultural biotechnology

## Abstract

This article explores the new developments and challenges of agricultural Gene Editing (GED) regulation in primarily nine countries of Latin America and the Caribbean (LAC) Region: Argentina, Bolivia, Brazil, Colombia, Guatemala, Honduras, Mexico, Paraguay and Peru. As Gene Editing technology develops, Latin America and the Caribbean regulatory regimes struggle to keep pace. Developers and regulators face challenges such as consumer perceptions, intellectual property, R&D funding (private and public), training, environmental and social impact, and access to domestic and international markets. Some Latin America and the Caribbean countries (e.g., Argentina) interpret existing legislation to promulgate regulations for biotechnology and Genetically Modified Organisms (GMOs), while others (e.g., Brazil and Honduras) have specific legislation for Genetically Modified Organisms. In both those cases, often a case-by-case approach is chosen to determine whether a Gene Editing organism is subject to Genetically Modified Organisms regulations or not. Other countries such as Peru have opted to ban the technology due to its perceived resemblance to transgenic Genetically Modified Organisms. After presenting the regulatory landscape for agricultural Gene Editing in Latin America and the Caribbean, this article addresses some of the differences and similarities across the region. Some countries have had more foresight and have dedicated resources to increase capacity and develop regulations (e.g., Brazil, Argentina, Colombia, Guatemala, Honduras, Mexico before 2018) while others struggle with bureaucratic limitations and partisanship of policymaking (e.g., Paraguay, Bolivia, Peru, Mexico after 2018). We propose that the differences and similarities between these regulatory regimes have emerged in part as a result of policy entrepreneurs (influential individuals actively involved in policy making) taking advantage of policy windows (opportunities for shaping policy and regulation). The third and remaining sections of this study discuss our main findings. Based on 41 semi structured interviews with regulators, scientists, product developers, NGOs and activists, we arrived at three main findings. First, there seems to be a consensus among most regulators interviewed that having harmonized regimes is a positive step to facilitate product development and deployment, leading to commercialization. Second, reducing bureaucracy (e.g., paper work) and increasing flexibility in regulation go hand in hand to expedite the acquisition of key lab materials required by developers in countries with less robust regimes such as Peru and Bolivia. Finally, developing public and private partnerships, fostering transparency, and increasing the involvement of marginalized groups may increase the legitimacy of Gene Editing regulation.

## 1 Introduction

GED is a new set of technologies that allow for targeted DNA modifications, with the most recent discovery being Clustered Regularly Interspaced Short Palindromic Repeats (CRISPR). CRISPR is a bacterial immune system that has been repurposed to be used in eukaryotic cells of animals and plants (Innovative Genomic Institute website, 2022). By combining CRISPR with the Cas9 protein, it is possible to make a cut in the DNA at a desired location and add, delete, or alter one or more nucleotides (Shukla-Jones, Friedrichs, and Winickoff, 2018). Gene Editing (GED) has increasingly attracted attention from scientists, policymakers, and regulators due to its potential uses in agriculture and human health. In the case of agriculture, it can be used to increase production, address climate change, and foster sustainability.

GED is known to be more precise than Genetic Modification (GM). Genetically Modified Organisms (GMOs) are created by inserting genes in random and multiple locations in the genome (Kuzma, 2018), with a low level of efficiency. GM is mostly used to create transgenic organisms, which require the insertion of foreign species’ DNA sequences in the modified organism. Instead, CRISPR can be used to create a cisgenic modification in which genes from within the same species are efficiently transferred through a single or set of base pair swap(s), or by performing a simple “knockout” or removing a sequence to alter an organism’s function or form (Kuzma, 2018). However, CRISPR can be also used to create transgenic organisms. This would happen if a donor template is provided that contains genes belonging to another species (or that are synthetic).

Due to these complexities, regulating GED, and CRISPR technology in particular, has become a challenge. Domestic and international regulatory bodies struggle to keep pace with emerging technologies such as GED, with many countries yet to commit to a path for regulation (Pixley et al., 2022). There is an important ongoing global debate about whether or not GED and GM should be regulated under the same frameworks. This is because GED may or may not involve the transitory introduction of foreign DNA sequences, may or may not result in transgenic products, and may or may not generate products that are different from those created through conventional breeding (Pixley et al., 2022). As a result, countries around the world have chosen different approaches on how to regulate GED technologies, with some implementing product-based regulations and others process-based (Entine et al., 2021).

For example, the United States, similarly to Argentina, does not have specific GMO or GED laws and instead uses current existing laws to promulgate GMOs regulations, focusing on the product rather than the method used to produce it (EPA website, 2017). On the other hand, the Court of Justice of the European Union (EU) determined in 2018 that organisms obtained through new mutagenesis techniques (including GED) are GMOs. As a result, GED products are currently still subject to the GMO-specific sets of regulations in the EU, which focus more on the process rather than the product (Van Der Meer et al., 2020). Concerning this issue, a recent article by industry authors Jenkins et al. argues that “process-based differential regulatory systems will also have a negative effect on the democratization of the technology” and that “regulation based on process will not advance common goals of nutrition, sustainability or consumer preference” (Jenkins et al., 2023). These authors focus on biotech companies’ growth, access to technology and regulatory burdens rather than food sovereignty challenges related to family farming which is common in some LAC countries.

In addition to these technical struggles, some contingents of advocacy groups and segments of the public continue to raise concerns about potential hazards of biotechnology products, adding to political and economic pressures that have shaped the design of regulatory regimes in countries such as those included in this article. For example, international environmental NGOs raise questions about potential hazards of GED and GMO products. While academics, regulators, and policymakers tend to regard these concerns as unscientific, concerns about toxicity and hazard potential of these products are still an important part of the landscape of innovation and potential deployment of GED products.

In this paper we focus on nine countries in the Latin America and the Caribbean (LAC) region. Drawing on concepts such as regulatory regimes, policy windows and policy entrepreneurs to describe and analyze the governance of agricultural GED in most of the LAC countries, we focus on the political dimensions that shape agricultural GED regulatory regimes by exploring how the domestic politics of nine LAC countries have created a heterogeneous patchwork of regulatory systems. Finally, we analyze the role of policy entrepreneurs in shaping policy discourse and regulatory regimes in the region.

## 2 Background: Governance of GED in LAC

### 2.1 Agriculture, biodiversity and innovation for agriculture in LAC

Accounting for more than 5% of GDP in over twenty countries and generally between 8% and 30% of employment (Morris et al., 2020; World Bank, 2023), primary agriculture, or cultivation of crops and breeding of livestock, remains a fundamental economic activity throughout the LAC region. This importance compounds further when ‘backward’ linkages to input sectors and ‘forward’ linkages to processing, transport, and retail sectors are considered. For example, while primary agriculture may compose only 3.8% of GDP in Chile, the compounded value-added share of GDP within the agri-food sector is estimated to reach 6.4% (Foster and Valdés, 2015). While experiencing sometimes volatile year-over-year fluctuations, the growth of agriculture (including fisheries) in the LAC region averages about 2.7% over the past 2 decades (OECD, 2019). Commodity trade is a particularly key export sector and source of foreign currency. Export products such as soybeans, pork, beef, maize, poultry, animal feed, sugar, coffee, fruits, and vegetables are drivers of LAC’s agricultural sector (OECD, 2019). The leading food exporter is Brazil (USD 79.3 billion in 2017), followed by Argentina (USD 35.0 billion), Mexico (USD 32.5 billion), Chile (USD 17 billion), Ecuador (USD 10.4 billion), and Peru (USD 8.8 billion) (OECD, 2019). During the past 2 decades, LAC’s agricultural trade surplus has increased, reaching USD 104.3 billion in 2017 (OECD, 2019).

As suggested by Roca et al., 2004, the LAC region is an important center of origin and diversity for different organisms that contribute to the world’s food security. However, LAC’s biodiversity is under pressure, since an estimated 12% of known wild plant and animal species are under threat of extinction (Brooks et al., 2016). The LAC region is the source of around 60% of the global terrestrial, freshwater and marine biodiversity (UNEP-WCMC, 2016). Another important dimension of LAC’s biodiversity is agrobiodiversity, which is defined as the genetic diversity of crop and non crop species (Morris et al., 2020), and is the result of the interactions between natural and human systems (Bioversity International, 2017).

Because of the abundance of its biodiversity, the LAC region has become a hub for innovation and technology development for agrifood systems. For instance, plant biotechnology has become relevant for increasing LAC’s production, economic and social growth (Gatica Arias, 2020). The same is true for GED in animals, where de Almeida Camargo and Pereira, (2022) argue that through gene editing local dairy cattle breeds, milk production can be increased, contributing to food security. Farmers in countries that allow GMOs may be able to produce more food per unit of land with fewer inputs, cultivate areas considered not suitable for agriculture and agrobiodiversity (Gatica Arias, 2020). However, as mentioned above, LAC’s NGOs and environmental groups are very likely to remain hesitant about these technologies.

Another critical dimension of the landscape of GED in LAC is how intellectual property rights (IPRs) intersect with agrobiodiversity conservation, the privatization of seeds and food security. According to Lokhandwala 2022, a relevant amount of the agrobiodiversity legal framework “lies within the intellectual property space”. Some authors have described how countries such as Argentina, Brazil and Paraguay have created favorable environments for biotechnology and IPR regimes (Newell, 2009; Filomeno, 2014). Intellectual property protections generally go hand in hand with maturity of biosafety regulations and are generally critical for private sector entrance into this space. The strength of IPR regimes have been used to categorize the maturity of biotechnology infrastructure in Latin American countries (Trigo et al., 2010). During the 1990s, most LAC countries adopted a neoliberal approach to agriculture due to severe financial problems that those countries were facing between the 1980s and the 1990s (Filomeno, 2014). Agriculture was seen as a strategic economic sector to continue paying foreign debt and achieve monetary stabilization. Intellectual property surrounding seeds and plant varietal development is quite controversial to some authors, while absolutely essential to others.

In a policy brief for the Inter-American Development Bank focused on Latin American biotechnology and the patent and licensing environment, Bagley (2021) notes the rapid growth of CRISPR patent families in the region and the importance of licensing structures to facilitate access to GED technologies. Foundational CRISPR-Cas9 patent holders are US-based, though (at the time of writing) the firm Corteva offers a bundle licensing approach for plant agriculture, namely,: 1) an internal only R&D license; 2) a commercial seeds and crop trait products license; 3) a commercial license for other (non-livestock) agricultural products (such as using a plant as a factory to produce therapeutic proteins); 4) a license to provide CRISPR-Cas9 services; and 5) a no-cost academic research license. The manner in which Latin American public and private sector entities are able to effectively access licenses and translate innovations to their populations will be extremely important in determining to what extent small scale producers will ultimately benefit from novel GED technologies.

### 2.2 Differences in LAC regulations for GED and GMOs

**FIGURE 1:**
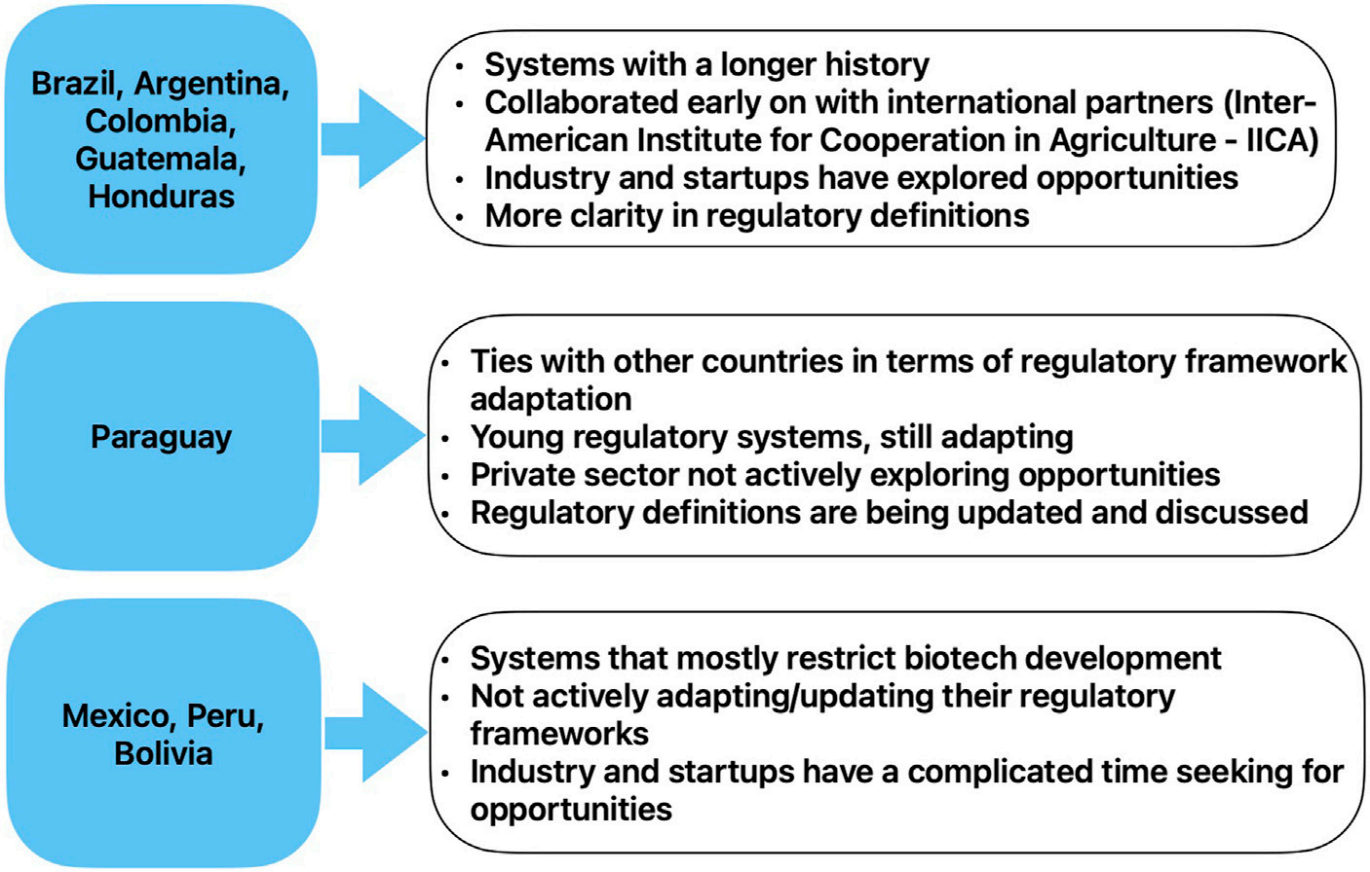
Broad illustration of some differences in LAC countries’ regulation and key policy aspects.

Since the emergence of the first generation of GMOs, the various countries of the region have taken different stances towards the applications of these technologies in agriculture, for multiple reasons illustrated in recent scholarship (for example, Roca et al., 2023). Some of these key differences are illustrated in Figure 1 below^1^.

Based on a recent study (Kuiken & Kuzma, 2021), it seems that countries with a longer history of biotechnology regulation such as Argentina, Colombia and Brazil are more open to innovation in general. On the other hand, countries such as Bolivia and Peru, that have a complicated history with biotechnology, seem to have more active and influential anti GMOs groups engaged in domestic politics. Countries such as Brazil and Argentina, which have a stronger culture of industries and startups, have more training opportunities compared to countries in which the private sector is not actively exploring opportunities to invest such as Paraguay (Zarate et al., 2023). Additionally, another important difference between countries’ regulations is the way in which they regulate cisgenic and transgenic organisms. In the next paragraph we explain more in detail these differences.

Due to its advanced regulatory approach to GED products, Argentina is perceived as being a leader in the region, at least according to most regulators and decision makers that we interviewed. Argentina is among the world’s top producers of GM crops, having approved 48 varieties for commercial use (Whelan and Lema, 2019) and has one of the oldest regulatory systems for biotechnology in LAC. In Argentina, the decision of whether GED products are subject to GMO regulations is taken by the National Advisory Commission for Agricultural Biotechnology (CONABIA, Comisión Nacional Asesora de Biotecnologia Agropecuaria) on a case-by-case basis according to the criteria of “novel combination of genetic material” (Kuiken & Kuzma, 2021). In particular, some varieties of GED crops most likely will not be considered GMOs in Argentina if the final product submitted to the authorities does not contain any transgenic DNA (Kuiken & Kuzma, 2021). GMO regulations were not altered and no exemptions were established for GED crops. It is important to mention that Argentina, like the United States, uses pre existing laws for the protection of the environment, food, animal health and plants to regulate GMOs and biotechnology in general. Argentinian regulators are considered global experts in this field. Based on our interviews, regulators and researchers in favor of promoting the use of GED in varietal development seem to share a desire for harmonizing regulations in the region based on the Argentinian model. Argentina is also one of the few countries that did not ratify the Cartagena Protocol on Biosafety (CPB) which is part of the Convention on Biological Diversity (Convention on Biological Diversity website, 2014). The CPB, which regulates the transboundary transfer of GMOs, was negotiated from 1996 to 2000 and entered in force in 2003 (Gupta and Falkner, 2006). This is important because other countries included in this study (Bolivia, Brazil, Colombia, Peru, Guatemala, Honduras, Mexico and Paraguay) have ratified it, therefore they need to implement new regulations to comply with these commitments (ECLAC website, 2003).

Brazil is another top country in the region for biotechnology crop production. It is actually the second in the world, with more that 100 GM events approved for consumption (Kuiken & Kuzma, 2021). Brazil is considered to have a robust regulatory capacity (Roca et al., 2023), with specific GMO regulations, and also ratified the CPB. The National Technical Commission on Biosafety (Comissão Técnica Nacional de Biossegurança - CTNBio) is in charge of determining whether a GED product is considered GMO or not on a case-by-case basis. Similarly to Argentina, if the GED product does not contain transgenes, it will most likely not be considered a GMO (Kuiken & Kuzma, 2021). On the other hand, in recent times, Mexico appears to have changed its regulatory stance towards GMOs despite being the 16th country in the world for biotechnology crops planted (Kuiken & Kuzma, 2021). In February 2023, the Mexican president issued a decree (President of the United Mexican States, 2023; Swanson and Qiu, 2023) which replaced the 2020 decree (President of the United Mexican States, 2020) that proposed a phased ban on all imports and approvals of GMO corn^2^. The new decree still requires a phased ban of glyphosate applications and GMO corn imports while at the same time requiring regulatory bodies to provide sustainable and culturally appropriate alternatives.

It is important to note that Mexico has not yet decided whether GED products will be considered GMOs or not under the Biosafety law, which currently regulates biotechnology related products (Kuiken & Kuzma, 2021). Similarly, Venezuela imposed a ban on GMO cultivation, as did Ecuador (constitutional prohibition) and Peru (GMO moratorium extended to 2030). Other countries in the region, such as Bolivia, do have regulations that govern the use, importation, and trade of GMOs as part of the CPB implementation process (Kuiken & Kuzma, 2021). However, there is the need to clarify and align the definitions contained in those laws in order to determine whether GED applications will be subject to the GMO legislation in Bolivia (Kuiken & Kuzma, 2021). Honduras also ratified the CPB and has regulated biotechnology products since 1998. Honduras is ranked 20th in the world for biotech crop planted area.

Although some countries took a very different stance on GED, the majority of the LAC region appears to share similar approaches to GED governance (Kuiken & Kuzma, 2021), with some GED products not being regulated as GMOs. However, Kuiken & Kuzma point to the uncertainty of how those differences will impact further negotiations at the global level, particularly within the CPB, where the EU and other reticent countries may hold strong influence. As it will be illustrated later in this article, the ratification of the CPB appears to have motivated policymakers in some LAC countries to expedite decision making on biotechnology and GED normatives.

This paper seeks to better understand the complexities of LAC’s regulatory landscape by focusing on the political and social dimensions of agricultural GED regulation across nine countries. LAC’s GED or GMOs legislation and policy often represents the outcome of multiple negotiations between parties such as governments, regulators, scientists and activists. The next section will explain the theoretical framework designed to understand how domestic and international politics have shaped agricultural GED and GMOs regulation in the region.

## 3 Theoretical framework: regulatory regimes, policy windows and policy entrepreneurs

### 3.1 Regulatory regimes

The framework of regulatory regimes will demonstrate how political standpoints and values around GED technology have shaped agricultural biotechnology regulation across different countries of the LAC region. Using a regulatory regime’s lens, we can explore “a range of risk-assessment techniques and policy-making approaches to distinguish the different scientific and bureaucratic practices, techniques, and cultures embodied in different fields of risk regulation” (Hood, 2001). This concept is useful to analyze the interests and motivations behind the integration or fragmentation of regulation, unwritten rules or statutory codes, inputs, processes and products, penalties or incentives, professional or cultural biases, rigor and preferred policy instruments, and biases towards market type incentives (Hood, 2001). We are interested in understanding how economic and political interests have shaped regulatory regimes in the LAC region. We focus on the mobilization of those interests across regulatory regimes rather than reducing decision making to maximizing actors’ own interests (Hayden, 2003).

Additionally, we are interested in investigating how regulatory regimes are shaped by policy processes. We follow the Weible Christopher, (2014) characterization of policy process research, defined as the “study of the interactions over time between public policy and its surrounding actors, events, and contexts, as well as the policy or policies’ outcomes”. In this framework, individuals and collectives can be considered actors that make decisions in the context of ambiguity. Events are defined as anticipated or unanticipated incidents, such as elections or crises. Contexts are considered to be shaped by socioeconomic, cultural, infrastructural and biophysical conditions, as well as institutions. According to Feldman, ambiguity is defined as “a state of having many ways of thinking about the same circumstances or phenomena” (Feldman, 1989). Ambiguity is understood as opposed to uncertainty, since the latter refers to the inability to predict an event and the former may be thought of as ambivalence (Zahariadis, 2014).

### 3.2 Policy entrepreneurs and policy windows

To understand the concept of policy windows as defined by Kingdon, it is necessary to explain the three streams of policy processes: problems, policies and politics. Problems are considered the issues that policymakers and citizens want addressed (Zahariadis, 2014), such as the COVID-19 pandemic. Policies are the ideas and plans developed by experts that compete to gain acceptance in policy networks (Zahariadis, 2014). Politics include the national mood (thinking along common lines and mood swings), pressure groups, and administrative or legislative turnover (Zahariadis, 2014). According to Kingdon, policy windows are “opportunities for advocates of proposals to push their best pet solutions, or to push attention to their special problems” (Kingdon, 2003). Those could include, for example, new elections, a negative event concerning a problem or the ratification of an international agreement.

Policy entrepreneurs are defined as those individuals or corporate actors that have the skill to identify and take advantage of policy windows to push for policies. As Zahariadis mentions, particular organizations can be considered policy entrepreneurs, not just their individual representatives. According to Zahariadis, policy entrepreneurs are more than mere advocates of solutions. Instead, they can be considered power brokers or coalition enablers. If the windows close, opportunities are lost and policy entrepreneurs must wait for the next opportunity to come along (Zahariadis, 2014). Additionally, they must be able to “attach problems to their solutions” and find those willing to be receptive to their ideas (Zahariadis, 2014).

### 3.3 Assessing regulatory regimes through an analysis of policy windows and policy entrepreneurs

**FIGURE 2:**
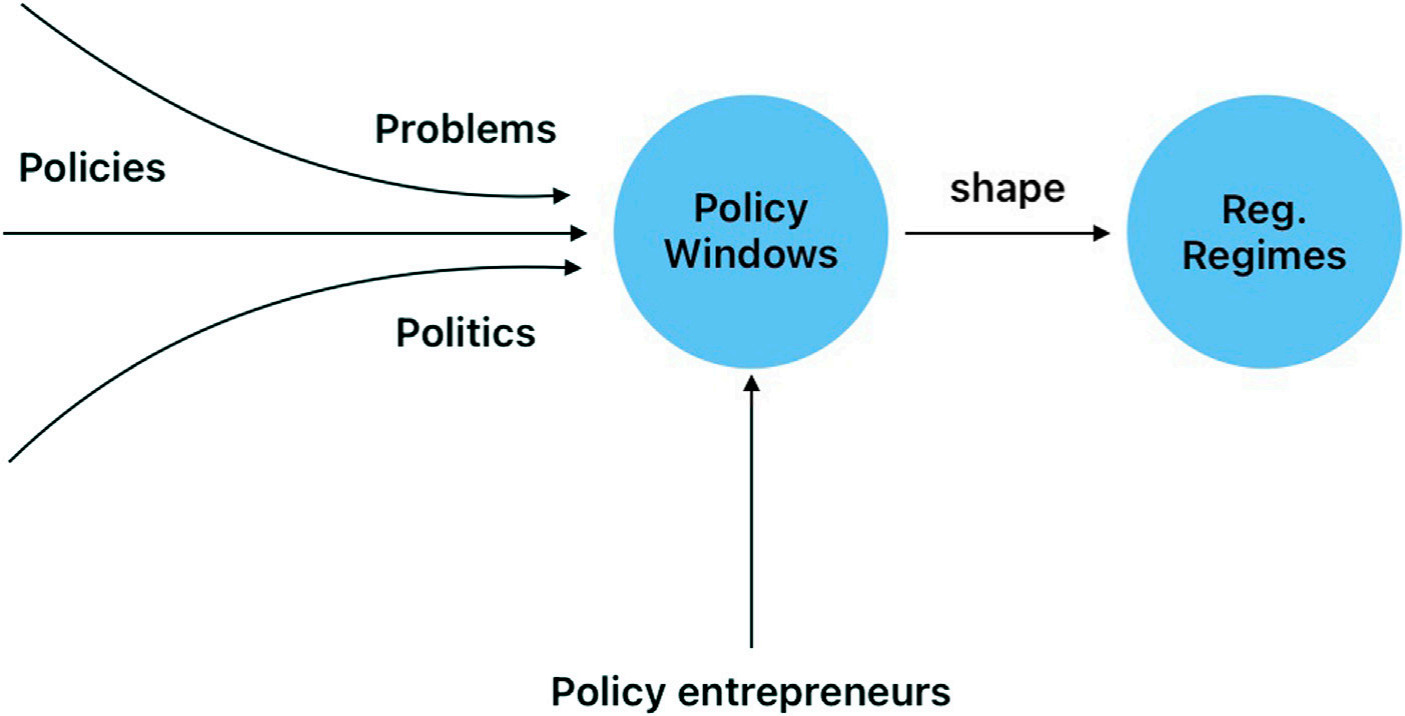
Policy entrepreneurs seize policy windows to shape regulatory regimes. Policy windows, according to Zahariadis, are the choices made when “the three streams are coupled or joined together at critical moments”.

We also seek to understand the policy processes that have positioned biotechnology and GED technologies as key drivers of LAC agriculture. We argue that agricultural GED regulatory regimes were shaped by multiple policy entrepreneurs who took advantage of key policy windows that facilitated or blocked the implementation of regulations in the LAC region (see Figure 2 for conceptual framework). These regulations and policies were often negotiated by most of the interviewees who participated in this study. Some relied on networks that facilitated agreements between governments, firms, and universities. Examples will be included in the results section of this paper.

Based on our theoretical framework, we examine how access to markets, legal definitions, formal and informal interactions^3^ (Atkinson, 1982; Diefenbach & Sillince, 2011) shape agricultural GED regulation in LAC. Identifying key differences and similarities across LAC’s regulatory regimes can contribute to the design and implementation of robust regulatory policies able to tackle key challenges such as increasing capacity, public engagement and public-private partnerships. Finally, this article also addresses a gap in the LAC literature about the governance of emerging technologies since we include the agency of the stakeholders as well as the societal system in which those actions take place.

## 4 Methods

We conducted 41 semi-structured interviews with experts and other stakeholders on the topic of GED for agriculture. The interviews were conducted over Zoom due to the ongoing COVID-19 pandemic. The data gathered to develop this paper was collected during a broader project carried out through a collaboration between the Inter-American Development Bank (IDB) and the North Carolina State University’s Genetic Engineering and Society (GES) Center, which main goals were to evaluate the current state of policies in the LAC region, analyze case studies to understand potential effects of policies directions, and identifying Bank investment priorities^4^. However, while carrying out the interviews analysis, we noticed the emergence of additional relevant information that sparked the idea for this paper and the subsequent analysis with the chosen theoretical frameworks.

The goal was to obtain a clear picture of the situation in the region, particularly concerning the regulatory frameworks in the different countries. The different criteria used to choose the interviewees are the following:

• **Country of origin**
• **Occupation.** The goal was that of interviewing individuals from different sectors, which include regulators, policymakers, researchers in public as well as private institutions and representatives of environmental groups and farming communities. Due to the scope of the original project, our research only included a small group of environmental activists and NGOs.
• **Position toward GED.** with the attempt to capture and reflect the different points of view in the region. As a result, the interviewees were either neutral, leaning pro or doubtful about the application of biotechnology and more specifically GED.

We performed multiple rounds of coding on the interview scripts and notes. First, we focused on the revision of the notes and scripts to identify adequate keywords that would capture the different topics that emerged from the interviews and that we deemed to be relevant for our study. Secondly, we checked that the keywords were used consistently and potentially expanded on additional complementary information. This phase was fundamental to identify some patterns and commonalities in the region concerning GED and biotechnology more broadly and helped us have a better understanding of the situation in the region. Afterwards, based on our understanding of the situation and the observed emerging patterns across the various interviews, we chose relevant existing theoretical frameworks through which to analyze the quotes, which are the ones introduced in the above sections. We therefore focused on some of those keywords that we thought were particularly important to our analysis and some of the corresponding quotes are going to be illustrated in the following section. Lastly, an audio and video revision has been carried out to confirm the accuracy of the selected quotes. The more we analyzed the interviews, the more our initial ideas evolved, and therefore some keywords’ original meaning was updated to reflect our new interpretation.

The software Taguette was used to perform the above mentioned coding, through which was possible to work collaboratively during this fundamental step of the analysis.

## 5 Results

### 5.1 Regulatory regimes: Politics create a landscape of heterogeneous regulatory systems

In Latin America there is a set of diverse regimes, with a tendency from countries with “developing” regulatory regimes to learn and harmonize with countries with more “developed” regimes. However, there is still a desire to maintain a certain degree of autonomy between LAC regulatory regimes. There are multiple reasons why those that are developing their regulatory regimes feel the need to harmonize and improve their regulations to “catch up” with those considered more advanced. The main one is the influence that regulations exercise on the ability to develop products and commercialize them, giving more options to develop ties between product developers, corporations and research institutions like universities. There are multiple interviewees that mention, for example, complications that include expensive processes for approval, problems at the border, problems at acquiring equipment and a worrying tendency of students to go abroad for both graduate education and employment.

As explained above, some LAC countries (e.g., Argentina) interpret existing legislation to promulgate regulations for biotechnology and GMOs, while others (e.g., Brazil and Honduras) have specific legislation for GMOs. In both those cases, often a case-by-case approach is chosen to determine whether a GED organism is subject to GMOs regulations or not. Other countries such as Peru have opted to ban the technology due to its perceived resemblance to transgenic GMOs. The Peruvian Congress decided to ban Living Modified Organisms (LMOs) through Law 29811 and Law 31111 (Peruvian Congress, 2011; Peruvian Congress, 2021). However, Peruvian legislation does not differentiate between transgenic GMO, LMOs or GED bans. At the same time, LAC’s regulatory regimes that established more relaxed pathways for non-transgenic GED include countries such as Honduras, Guatemala, Colombia, Brazil and Argentina.

Interviewees affiliated with NGOs tend to argue that there are similarities between those technologies, representing a “GMO 2.0” in terms of social and environmental impacts. In this case, GED is rejected due to its perceived similarity to GMOs. Even though some regulators and policymakers may raise concerns about this transition, we argue that this debate and its regulatory and societal implications can be transferred to GED governance when LAC countries adopt GED technologies and enforce robust regulations. If (non-transgenic) GED products are regulated the same way as transgenic products, then it becomes important to revisit the way in which perception, legal definitions and access to markets change or remain the same.

#### 5.1.1 Guatemala and Honduras: regulation shaped by Free Trade Agreements and customs unions

Both countries’ regulations were primarily shaped by the Free Trade Agreement that facilitated the development of biotech regulations and expedited product development, and have since evolved with the adoption of Customs Union agreements. The Dominican Republic-Central America-United States Free Trade Agreement (CAFTA-DR) was signed by the United States of America, the Dominican Republic, Costa Rica, El Salvador, Guatemala, Honduras, and Nicaragua in 2004 and went into effect in Guatemala in 2006 (International Trade Administration website, 2022). Since the late 1990s, Honduras has been a country considered an ideal destination for GMOs supporters. At that time, Guatemala was not motivated to approve GMOs because they considered themselves the center of origin of maize:

Since we began with the regulations in the late 90s, people used to say, “if you’d like to import GMOs, go to Honduras”. That was the gate to get into Central America”. The position of other countries, specifically Guatemala, was that they considered themselves the center of origin of maize. They were not eager to approve GMOs, at least for corn. For a long time, we were the only ones. In 2016/17 came this commercial agreement, called something like a customs agreement between Guatemala and Honduras. In 2017, I went there to advise their officials and academics. With our advice and training, they designed their legislation in the same terms as ours.

According to one interviewee, the USDA wanted the customs union agreement to become a reality and pushed for it: “I think the people in Guatemala, internally, did not agree. Some said we must sustain our claim to be a center of diversity and stuff like that, but others said that we need to catch up with the rest of the world. USDA put a policy in place, paid our trip to Guatemala, and promoted the meeting there. USDA wanted the agreement to come into place”.

In May 2016, the Guatemalan Congress approved the customs union with Honduras which allowed the “free movement of people and goods between the two countries” (International Trade Administration website, 2022). A year later, both countries carried out the first stage of the customs union process right after addressing regulatory, technical and administrative procedures. In 2019, these countries approved a “harmonized biotechnology and biosafety regulation” for GED plants, which is considered the first in Central America (USDA, 2020). It is important to note that Guatemala had a moratorium in place with the previous regulation that “did not allow for the commercial production of GED plants” (USDA, 2020). According to one interviewee, the new regulation offer simplified procedures:

In Guatemala, we are open to edited products. We handle them as conventional. A form is filled out, and in a week the user is informed, and an authorization is given without a time limit. We have an agricultural biosafety committee, but it is only for genetically modified organisms and commercial authorizations. We have a simplified procedure, which is common in Central America.

#### 5.1.2 Argentina and Colombia: learning from neighbors

Argentina is considered to have one of the most developed regulatory frameworks in Latin America. While Argentina did not ratify the CPB, its regulations include definitions that are compatible with the CPB (Kuiken & Kuzma, 2021). As one interviewee argues, the problem was its *non-technical* considerations:

Our regulations are completely in line with the technical part of Cartagena protocol. The safety assessment and the definition of a GMO. All of that has been in the regulation from the start. On the technical side, Argentina has always been in compliance with the protocol. The problem that the country had was with the non-technical part like liability and redress, socioeconomic considerations.

Another feature of Argentina’s regulatory regime is the way in which neighbor countries “mimic” its regulation. This happens with the way in which the definition of GMOs is shared: “We came to this strange situation in which our approach can be mimicked by other countries in Latin America or in other regions because they use the same definition [LMO definition]”. The LMO (living modified organism) definition is used in the CPB (Whelan and Lema, 2015). Definitions are important for regulators and risk analysts. According to an interviewee, there is a desire to harmonize regulations and achieve a synchronization of approvals. Ideally, harmonized regulations could reduce costs and spread benefits easily and effectively.

One of the LAC countries that has learned from Argentina is Colombia. Interviewees recognize the differences across both regulatory regimes. However, they consider that there is a need to learn from others. In particular, an interviewee expressed the importance of “catching up” to others and that regulation in Colombia should not inhibit research:

The pressure [to “catch up”] came from Brazil and Argentina, which also had regulatory frameworks. Colombia decided that if they did it already, we must do it. For plants, the institution in charge is ICA, the equivalent of USDA. It was good to have ICA doing the regulatory framework for plants, as they have a very long experience with plants.

The Instituto Colombiano Agropecuario (ICA) was created in 1962 as an agency of the Colombian Ministry of Agriculture and Rural Development (ICA website, 2022). ICA participated in the negotiations of bilateral or multilateral sanitary and phytosanitary agreements (ICA website, 2022). Other research centers such as the International Center for Tropical Agriculture (CIAT), have a longstanding reputation in Colombia. According to one interviewee, there is a need to think in advance about the technology and its relationship with the regulatory landscape. Researchers should be proactive and “bring regulators to the lab”.

#### 5.1.3 Brazil: overcoming an embroiled regulation

Brazil has a regulatory regime that is slightly different from other countries in Latin America. Much like the discourse in Argentina, there was a debate around the CPB and the CBD. Nevertheless, in terms of its regulatory framework, interviewees considered that it was embroiled until 2005:

There were a lot of missed opportunities because the regulatory scenario was really embroiled, completely embroiled in the beginning until 2005 [ … ] And then it changed for the better. But still there was a long way to go. Now I think regulation is mature, and is ready to receive applications from universities, from small companies, from startups. Now with gene editing, we can exclude some products from assessment. An easier and cheaper assessment. Now the field is open for biotechnology, but it was not like that 10 or 12 years ago.

In 2016, Brazil approved what is known as the Normative 16. Before this normative, it was not clear from a regulatory standpoint if products would be considered GM or not. One interviewee mentioned that this normative was created to facilitate companies to invest:

In 2015, CTNBio [Brazilian National Technical Commission on Biosafety] decided to create a working group to start thinking about creating a normative to regulate gene editing in Brazil. At that time Argentina, the United States, Canada, other countries like Chile, Colombia were thinking about creating something related to gene editing. The process became clear to us, even for the government. We should follow the same pathway, the same standards to create this process to give chances to not just big companies to be on the market. At least in our view, regulation, the whole process of regulation of GM products is so expensive, and so complicated and so different in different countries that create a difficult environment.

Similar to Argentina, precision with definitions is important in Brazil. Some interviewees mentioned that the wording of Normative 16 is in accordance with the 2005 Law and the CPB, and that this measure does not intend to modify Brazilian Law. As with the regulations designed for Guatemala and Honduras, the goal of Normative 16 was to save time and reduce costs. CTNBio regulates technologies on a case-by-case process and interviewees claim that, under this normative, it takes approximately between two to 3 years to commercialize a product in the market. Compared to the transgenic products, regulatory compliance costs are considered much lower for GED products. Lastly, Normative 16 was also developed to reduce bureaucracy that, as interviewees mentioned, causes ‘asynchronous approvals’ in which there is a lag between the timing of cultivation approval and import approval.

#### 5.1.4 Mexico, Peru and Bolivia: regulation shaped by politics

This group of countries’ regimes seem to be influenced by domestic politics and environmental activist networks. The governments of Mexico, Bolivia and Peru, as well as civil society and indigenous groups, have opposed the development of transgenics and biotechnology. In the case of Mexico, regulations seem contradictory and confusing to some interviewees:

There are contradictions, inexplicable moratoriums, there are irreconcilable points, of the agencies that protect the environment and those that promote the agricultural sector. There is little understanding between what the law says and what officials do. The law had many heated debates, which reconciled biosafety from gradual, experimental liberations, pilot tests, and commercial evidence, which obeys international principles.

Additionally, interviewees mentioned that the regulation has not been modified since 2009 except for corn. To this date, this means that Mexican Law cannot be applied to products developed with GED. According to one interviewee, the regulation in place for agricultural developments is the National Biosafety Law from 2005:

The legal framework in place for any agricultural development in GMO, follows the 2005 national law of biosafety. This law gives the general framework. It has several considerations, [such as] the regulatory aspects of biotechnology, health, forests, etc. What the law regulates is the product and the process in Mexico. Despite its known or unknown implications, you must go to several stages of development.

Interviewees mentioned that before moving towards a commercial release, researchers first need to design an experimental research and a pilot stage. If these products are for human consumption, the Ministry of Health is involved. Additionally, some products are allowed to be released while others are not:

The policy in terms of GMOs, commercial, research, field release for cotton is in place. Maize is not released in Mexico [ … ]. We used to have a permit for soybeans, but currently there are no permits. Transgenics are not well received, it is a big debate.

In the case of Peru, an ongoing GMO moratorium affects the development of biotechnology. The original moratorium, approved in 2011, was established with considerable support from civil society organizations opposed to agricultural biotechnology. Law 29811 stipulated a 10 years moratorium that sought to prevent the entry and production of LMOs for “cultivation or breeding purposes, including aquatic ones, to be released into the environment” (Peruvian Congress, 2011). Through Law 31111, in 2021, the Peruvian Congress approved the extension of the moratorium until 2035 (Peruvian Congress, 2021). According to an interviewee, the Executive instead aimed for a new Biosafety Law and a shorter moratorium. Additionally, according to the same interviewee, previous regulations approved in 2002 for LMOs were not implemented efficiently.

The position of the Peruvian Ministry of Environment on this matter was not seriously considered by the Peruvian Congress in 2021. Interviewees argue that this was a political decision that had both positive and negative impacts:

The Ministry of Environment issued its opinions, its pros and cons, which were not taken into account in this commission. Finally, they approved it for 15 years even though other projects proposed 10 years. It was a political decision. The positive impacts of it were the availability of greater resources, fostering agrobiodiversity, and family agriculture. The negative impact was the restriction of a technology, to improve the productivity of small farmers.

A common problem in Peru and Bolivia is getting reagents and other key laboratory materials through customs. Interviewees from both countries mentioned different challenges such as substantial paperwork (Peru) and an association with illegal activities such as drug trafficking (Bolivia):

There are restrictions regarding the production of drugs. The law pursues you. They will come to your laboratory to see that you used your reagent, even if it is pedagogically. Importation (of reagents) is very bureaucratic. They must make a report on the health and food implications. Import is expensive, and many legal processes are involved.

GMOs were banned in Bolivia from 1997 to 2005 due to the pressure from the environmental groups. According to one interviewee, even with this pressure it was possible to publish a supreme decree in which a shorter procedure was included for risk assessment:

There was a moratorium on all GMO events from 1997 to 2005 due to this pressure from the environmental groups that persists. But there are scientists and academics that talk about benefits. Even while developing regulations, we talk about the importance of science over the economic, social, and cultural fears. Bolivia is trying this, starting from the producers, and I think that a positive sign in the country’s politics came in 2018. The government of Morales was very close to regulating and using biotech, but then it published a supreme decree where a shorter procedure was introduced to evaluate the risks.

#### 5.1.5 Paraguay: an evolving regulatory regime

Between 2005 and 2012 there was an official restriction from the Paraguayan government to release new transgenic crops. This changed in 2012, when the Paraguayan government was open to the release of new events and transgenic crops such as cotton:

It started with the acceleration of commercialization, first with transgenics. Then between 2005 and 2012 there was an official restriction with the release of new transgenic crops when I had to go in that direction. There was political pressure to avoid the release of new events; there were not many transgenics at that time. In 2012, new government policies started to focus on the analysis and to be open to the release of new events and transgenic crops. Cotton was released, others in the same way because we had issues with Argentina that had releases in 2016.

In 2019, the Paraguayan government published a resolution for crops developed using GED and other new breeding techniques (Genetic Literacy Project, 2020). Additionally, Paraguay issued a joint statement alongside twelve other countries including Argentina, Brazil, Australia and the United States to the World Trade Organization supporting relaxed regulations for GED (WTO, 2020). According to an interviewee, the pandemic slowed down this process:

Then we had the pandemic that has slowed and stopped what occurs in regulatory systems [ … ] Requests for microorganisms have increased, but I do not know if they have been regulated by GED regulation.

### 5.2 Policy entrepreneurs and policy windows in the LAC region

We consider environmental activist groups and grassroots organizations as policy entrepreneurs. Those groups are defined as public interest groups, which may be understood as counterpoints to the self-interest groups such as industry (Kingdon, 2003). In most cases, these groups are mobilized internationally through advocacy networks in a process called “transnational advocacy” (Keck and Kathryn, 1998). This term refers to the situation in which states are unresponsive to the demands of their citizens, and therefore activists may seek the support of international allies. Their main goal is to push public attention to issues such as food sovereignty, indigenous rights and agroecology. We also consider regulators, risk analysts, developers and scientists as policy entrepreneurs if they have actively influenced the adoption or rejection of new GED regulations.

In this study we aim to understand how these different policy entrepreneurs took advantage of policy windows to reconfigure agricultural GED regimes. We are paying attention to how domestic affairs (national legislation, elections, agriculture and environmental policies) and internationally-driven events (trade agreements, ratification of international agreements, partnerships) constitute policy windows.

Public interest groups often aim to establish transnational advocacy networks to increase their relevance and the resources available to them, primarily blocking the development of regulations that allow applications of GED in agriculture while pushing for alternatives. On the other hand, the other set of policy entrepreneurs (regulators, policymakers, risk assessment experts) primarily focus on pushing for the development of regulations that would allow the use of GED in agriculture with an eye on harmonization around the LAC region. For example, policy entrepreneurs have taken advantage of international agreements to steer legislation in favor of GED technologies, such as in the case of Guatemala and Honduras.

#### 5.2.1 Domestic policy windows

An example of a negative domestic event that opened a policy window that was seized by NGOs and civil society representatives in Peru is the finding of GM corn in the environment. According to a Peruvian interviewee, in 2008 a report identified the presence of GM yellow corn in the Barranca valley (Gutierrez-Rosati, 2008). This interviewee mentioned that this report triggered the establishment of the current moratorium:

In 2008 due to a report of transgenics in the environment, civil society organizations, farmers and social movements declared the country free of transgenics. In 2011, after a few years, Ollanta Humala [former Peruvian president], promulgated [the GMO moratorium] in December 2011, valid for 10 years.

In Bolivia, small farmers, concerned with GM crop imports from Argentina, triggered a shift inside the government that opened the possibility to discuss the matter:

So these small farmers said “why did our government import corn from Argentina when we can produce our own corn in Bolivia, with our techniques, our tastes”. This caused some shift inside the government and this allowed an opening towards this discussion.

Therefore, some small producers that are in favor of GED and GMOs crops, particularly from the Santa Cruz region in Bolivia, are trying to act as policy entrepreneurs and seize the policy window to push to have clear regulations about GED:

It is important to say that even the smaller producer is convinced of the benefits of the biotech and is open to the new tech like CRISPR because they know they can get more benefits. So, they are trying to influence the current government to make sure that these technologies have clear regulations and that can help the producers to produce more, and more sustainable agriculture. Small producers are very important in Bolivia.

As explained before, national elections are usually perceived as a policy window. In fact, an interviewee from Honduras mentioned it was an awaited event to push for new regulations:

It is a matter of time. The regulation that comes with the law. It is a matter of time [ … ] We have a lot of pressure to do it [ … ] Congressional elections coming in November, there is a chance to publish this regulation.

In Honduras, it seems that those that acted as policy entrepreneurs belong to the private sector, who apparently influenced the Ministry of Agriculture:

The Ministry of Agriculture and private entrepreneurs pushed for inclusion of regulatory updates for GED. When they wanted to import new technology they would not have any problem with it. The Ministry of Agriculture has focused on this issue, but on suggestions from the private sector.

#### 5.2.2 Internationally-driven policy windows

An example of policy windows that opened thanks to internationally-driven events is described by an interviewee from Guatemala, who explained that the Customs Union Agreement helped to pass regulations for biotechnologies, which was supported by the private sector and academia:

The regulations [for biotechnology] would not have passed without the Free Trade [i.e., Customs Union] Agreement […] It was fundamental, it would not have been possible if it had not been done under that premise, it allows the regulation to be maintained […] The Ministry of Economy negotiates the treaties. The Ministry of Agriculture carries out the technical proposals. There was support from businessmen, the private sector, and the academic sector.

Interviewees mentioned that the ratification of the CPB and the subsequent requirement to pass national regulations to comply with it pressured the different governments to act further in the biotechnology sector. The CPB’s influence is tied to international trade imperatives mediated by domestic politics (Gupta and Falkner, 2006). However, as Gupta and Falkner suggest, the flexible interpretation of the CPB has motivated countries to choose their own paths in biosafety policies. For example, one interviewee mentioned that due to the ratification of the CPB, the decision making moved faster:

When the Cartagena Protocol was ratified, the law established that the entity in charge and the focal point was not the Ministry of Agriculture but the Ministry of the Environment. This made things move very quickly for 2 years because not everything had to go to the national biotechnology commission.

Interviewees from both Honduras and Guatemala also mention the Inter-American Institute for Cooperation in Agriculture (IICA) and its role in supporting the development of regulations and policies in both those countries. IICA is an international agency based in Costa Rica specialized in agriculture of the Inter-American System; their goal is to support the Member States in agricultural development and rural wellbeing (IICA website, 2022). One interviewee explained that IICA respects local systems and regulations, working both with stakeholders in favor and against GMOs, as they aim at providing them with information about the regulations that exist elsewhere to make informed decisions.

Based on our interviews, it seems that IICA also acted as a policy entrepreneur in the LAC region. In a sense, it acts as a mediator between governments and other economic and social actors involved in LAC agriculture and rural living (IICA website, 2022). Most of our interviews highlight IICA’s role in fostering ties between academia and regulators. For example, an interviewee from Guatemala mentions that IICA supported academics and authorities in advancing and harmonizing regulations. The same interviewee mentioned that some individual consultants were particularly active during this process.

#### 5.2.3 Pressure from international environmentalists: the perceived European influence over LAC countries

We note an interesting pattern in our data: the repeated references to the influence of international environmental organizations in LAC, primarily from Europe. Most of the interviewees are concerned with the perceived European influence. One interviewee even described the European Union as “the worst enemy”. An example that shows these widespread concerns is the quote below from a Bolivian interviewee:

In Bolivia, as well as in other Latin American countries, we suffer from interference from European environmental organizations with strong investments and [ … ] (they) introduce a lot of fear over not only the production of transgenic crops but also on the consumption of these products. They also introduced fear [ … ] these new technologies, like CRISPR, can potentially change the genome of humans that consume products obtained with CRISPR.

This interviewee continued by mentioning the perceived presence of a heavily financed environmentalism in Bolivia, with influence in the civil society but also in the State. The interviewee frames the interference as a problem that affects not only the current government but also previous ones. The interviewee argues that the trend is observable since the 2000s, mentioning a moratorium on all the GMO events from 1997 to 2005 due to this pressure from the environmental groups.

Multiple interviewees also articulated that those environmental groups do not have strong local roots, primarily being foreign organizations. This concept is represented in the following quote from an interviewee from Brazil:

Activist groups are always international [ … ] Very rarely we saw small farmers, or agriculture, or students connected with those movements. It was not a spontaneous presence, it was organized internationally. The same issues were brought back, same questions were brought to other countries.

One aspect that caught our attention was the discrepancy between how those European organizations are perceived by those interviewees, that primarily come from a policy or academic background, and the activists themselves. The former, who tend to be in favor of GED, appear to support the idea that the European organizations are behind the anti-GMO network in the LAC region, particularly in some countries (for example, Mexico, Bolivia and Peru). Interviewees suggested that local opinions are in fact influenced by anti-GMO organizations’ agendas.

However, the activists themselves described a strong local presence, referring to specific events that motivated the formation of local organizations. Additionally, from the interviews it emerged that while they do actively seek international support, both from Europe and from other countries in the LAC region, international support is a strategy to gain additional strength and help in advocating for their domestic issues. As this interviewee from Brazil mentions:

People from outside, global vision, internationalize the fight and the hope, to have another dimension. Without the external pressure, the situation would be worse. It is an additional help for our internal fights.

The same interviewee adds that their organization has activities in the macroregion, collaborating with individuals from other countries in the region including Peru, Chile, Argentina, Paraguay and Nicaragua.

As explained above, the need to network with international organizations (i.e., transnational advocacy), appears to be triggered by the fact that the interviewees feel that their concerns are often not properly addressed in the decision making process concerning GED/GMO applications in agriculture. Some of those concerns include potential health hazards, accessibility to the technology and ultimately for whom GED is going to be more beneficial. For example, a shared concern is the possibility that the technology will primarily favor the big corporations rather than smallholders, as explained by an interviewee from Paraguay:

Our question is, how will this benefit us? There is incredible technological development, but how can this development benefit these poor people? How can we protect our seeds? And how can we access this? Technology needs to develop us as much as it develops the big companies. This is our fear: develop technology, but in the hands of big companies, and not in favor of small indigenous farmers.

Similarly, an interviewee from Brazil argued that big corporations are supported by the government, having a considerable amount of food exported while at the same time “local people continue to starve”. This interviewee felt that the public should benefit from these technologies:

The government does nothing for the farmers, but supports the big agribusiness. There is not even a single incentive for the farmers’ production. There is nothing positive for women farmers in the Bolsonaro’s government. [ … ] There are a lot of people that starve. [ … ] If people from Brazil would benefit then yes, but it is all exported.

As mentioned at the beginning of the paper, although the LAC region is quite resource rich, there still persist food insecurity and poverty. GED could be used to increase agricultural production. However, the interviewed members of local environmental and farmers organizations fear that this increased crop production would primarily be exported, rather than used to address domestic food insecurity.

[In Paraguay], big agrobusiness produce to export. At the roots of poverty and the death of our people. We are not against development, but we are against the exploitation of nature [ … ] privatization of seeds that are a heritage of our people. Today it is more and more in the hands of companies.

As highlighted in the above quote, it is important to note that activists are not necessarily against the use of technologies such as GED. They consider them to provide opportunities for development if used in a transparent and fair manner.

## 6 Discussion

The development of agricultural GED regulation requires the involvement of stakeholders familiar with science, legislation, policy and public engagement, as well as keeping pace with evolving domestic and international trade agreements and other treaties. While fostering deliberation around these technologies may appear to hinder technology adoption (Kuiken et al., 2021), it reflects the negotiations undertaken by regulators, product developers, and social movements around food sovereignty. Often, these negotiations are not known or explored, and thus deliberation may turn into tension or conflict. As Kuiken and Kuzma (2021) suggest, it is important to consider where GED is going to be implemented, whether there are markets for biotechnology products, and whether the public approves, trusts, and has equitable access. Kuiken et al. (2021) consider that the international governance of GED, in particular CRISPR, will play a crucial role in food and agricultural markets.

This paper showed that the differences between agricultural GED regulatory regimes across the LAC region can be explained partially by the variety of ways in which policy entrepreneurs (the different interest groups represented by academics, industry, ministries, congress, regulators, NGOs, scientists) have influenced agricultural GED regulation through policy windows.

The complexity of the stakeholder landscape and the dynamic political cultures we have studied contribute to heterogeneous agricultural GED regulatory regimes. We have shown this through the analysis of our interviews, where we particularly focused on domestically and internationally driven policy windows, as well as on the political pressure exerted by networks of activists and NGOs. These stakeholders are able to shape regulation through political interactions in formal and informal spaces. The role of policy entrepreneurs, especially in our definition that includes transnational advocacy networks, is a critical and potentially overlooked causal factor in the complexity of the regulatory landscape. In other words, without careful consideration of a wider range of policy entrepreneurs, we may be missing important context for what gives rise to different policy regimes.

How might lessons from LAC transfer to other geographies? What ideas might translate? Which are specific to the LAC region?

Additionally, little is known about the perspectives of growers and potential end users of GED technologies in the region. In regulatory cultures that emphasize being scientific or evidence-based, we would like to highlight that systematic social science data is indeed evidence and there are clear gaps where social science data is needed, particularly in the context of growers and historically marginalized groups. Our research only included a small group of environmental activists and NGOs. While these preliminary conclusions suggest compelling complexity, our research would be bolstered by additional research particularly in local communities.

In our case, public interest groups constituted by the NGOs primarily work to block the development of regulations that would allow the use of GED/GMOs in agriculture, while pushing for alternatives (e.g., agroecology). On the other hand, industry and academia tend to advocate for harmonious, comprehensible, and permissible regulations that would allow the application and diffusion of GED/GMO products which it is believed would foster R&D and overall economic growth. The identified groups, or groups that we call policy entrepreneurs, invest a considerable amount of resources like time, energy, reputation and money in the attempt to see their solutions transformed into regulations. It is important to note that this influence was undertaken also through formal and informal negotiations. In Guatemala and Honduras, informal negotiations (between the country’s ministries and external organizations like IICA, with particularly active consultants) led to the current regulatory framework. In countries such as Peru, negotiations in formal spaces such as the Congress led to the current GMO moratorium.

As mentioned by Hood, (2001), an analysis of regulatory regimes may explain the integration or fragmentation of regulatory frameworks, as well as the policy instruments and potential biases towards market type incentives. For instance, the regimes of countries like Argentina, Honduras, Guatemala, Colombia and Brazil are designed to support a stronger relationship with external markets and are generally more open about applications of the GED technology in agriculture to increase production. These regimes tend to foster public engagement as well as harmonization of regulatory frameworks across LAC. On the other hand, regimes of countries such as Peru, Bolivia, and Mexico generally restrict agricultural GED applications primarily due to its perceived resemblance to GMOs. Stakeholders that oppose agricultural GED have pressured their governments with a stated purpose to protect family agriculture and food sovereignty.

At the same time, there are common issues across the LAC region in terms of the definitions used in regulation. Since legislation changes across countries, similar definitions of what is and what is not a GED product may impact product development and regulation. In order to move towards a more harmonized regulatory framework across the region, the concerns of public interest groups (environmental NGOs, farmers and indigenous communities organizations) need to be taken into account. This could be achieved by providing clear and transparent information about the differences with GMOs, how GED works, the safety of these technologies and how these will benefit them.

Although we support the need for science (particularly molecular biology and risk evaluations) to inform regulations, we also believe that dismissing social science data may be detrimental to achieving the goal of regulation development and harmonization in LAC countries. The general belief that the anti-GMO movement in the region was born due to international influence, particularly from the European Union, appears to conflict with local organizations’ self-description of their origins and scope. The interviewed activists explained the local roots of their organizations, adding that they are not necessarily against GED and GMOs, but they are concerned with the health impacts of products obtained through these technologies, and question the benefits for small producers. Therefore, we believe that there is the need to collect sound social science data on those local groups to be included in the body of knowledge considered to formulate regulations. The conversation on those technologies should be broadened to potentially positively or negatively impacted, marginalized communities to obtain a complete picture of the political landscape in the region.

## Data Availability

The raw data supporting the conclusion of this article will be made available by the authors, without undue reservation.

## Footnotes

1 It is important to note that Mexico’s current administration has a different attitude towards GMO and GED regulation. Additionally, it is only since 2016 that Guatemala and Honduras have a harmonized regulation driven by the Customs Union Agreement. In our results section we explain more in detail the development of GED regulations in these countries.

2 The current government has not approved any new GMOs since May 2018, rejecting additional permits to plant GMO cotton in 2019 although previously approved (Kuiken & Kuzma, 2021; Roca et al., 2023).

3 Formal interactions are interactions in formal spaces such as those defined by internal policies and norms, or legislation. For instance, Congress is a formal space for passing regulation. Informal spaces are the personal interactions that individual actors or organizations have to negotiate between them without necessarily moving to a formal space.

4 For further information, please visit the project’s website at the following link: https://research.ncsu.edu/ges/research/idb-crispr/#top
